# Insertion/deletion polymorphism of angiotensin-converting enzyme and chronic obstructive pulmonary disease: A case-control study on north Indian population

**DOI:** 10.22099/mbrc.2019.34904.1438

**Published:** 2019-12

**Authors:** Nikhil Kirtipal, Hitender Thakur, Ranbir Chander Sobti

**Affiliations:** 1 Department of Biotechnology, Panjab University, Chandigarh-160014, India; 2 Department of Biotechnology, SUSCET, Tangori, Punjab-140306, India

**Keywords:** ACE, COPD, Polymorphism, Genotype

## Abstract

This research aimed to explore the *ACE* (insertion/deletion) gene association as key factor for chronic obstructive pulmonary disease (COPD) development in north Indian population. A total of 200 clinically diagnosed patients with COPD were selected against 200 healthy individuals. Genetic variations of *ACE* (insertion/deletion) were evaluated by using polymerase chain reaction techniques. Smoker showed higher risk of COPD (OR=1.67, 95% CI=1.12-2.48, P=0.012). Present results revealed the positive association between the DD genotype and the risk of COPD (OR= 2.14, 95% CI=1.22-3.78, P=0.006). Among smokers, DD genotype showed statistically significant association with increased risk of COPD (OR=3.10, 95% CI= 1.50-6.47, P=0.001).

## INTRODUCTION

Chronic obstructive pulmonary disease (COPD), is defined as an inflammation in the respiratory tract. In addition to participation of metabolic and cytokine gene in COPD development, *ACE *(Angiotensin-Converting Enzyme) gene was also suggested as essential factor responsible for the progression of pulmonary hypertension which may leads to COPD [1].

The *ACE *gene which is located at chromosome 17 (17q23 region) was discovered with insertion I allele or deletion D allele polymorphism of 287 base-pair in *Alu* repeats nonsense DNA domain [2]. The association between* ACE* DD genotype and pulmonary hypertension during exercise in COPD patients has been reported. This excessive pulmonary hypertension due to exercise was suggested in the disturbance of oxygen passage into the tissues. Significant co-relation between the *ACE* DD genotype and COPD risk with pulmonary hypertension amongst the Asian people by meta-analysis was established [3]. The involvement of renin in angiotensinogen conversion into angiotensin I was suggested as the first part for renin-angiotensin system (RAS) activation [4]. However, association of the renin-angiotensin system (RAS) with COPD pathophysiology was also documented. Moreover, retrospective studies reported that ACE (angiotensin converting enzyme) inhibitors and RAS reduce the mortality in COPD patients. Thereof, it is seen as promising therapeutic approach for COPD [5, 6]. In this study, we attempted to seek out the relationship between *ACE* I/D gene variations and COPD north region population of Indian subcontinent. 

## MATERIALS AND METHODS

**Patients and questionnaire assessment** **: **This study was conducted on total of 400 subjects including 200 patients pathologically diagnosed with COPD against 200 healthy controls. All participants were recruited from the hospitals nearby region of Himachal Pradesh and Chandigarh in the north India. Each participants detailed inform consent was taken before the study. Set of population was provided the detailed inform consent of the patients during this study. 

In our study, COPD patients were defined as participations with (i) chronic respiratory symptoms and signs such as breathlessness, cough and wheezing, (ii) less than 80% predicted forced expiratory volume in 1s (FEV1), (iii) FEV1/forced ratio for vital capacity (FVC)≤0.7, (iv) patients in age range of 19-65 years were included for positive COPD test and (iv) irrespective of smoking, tobacco intake, alcohol consumption status. Additionally, healthy smoker controls were also subjected to chest radiography to rule out any abnormal pulmonary function. Whereas, participations were excluded with (i) medical history of asthma, (ii) malignancy such as lung cancer or autoimmune disease was also considered forthe test group to avoid spurious results and (iii) individuals with smoking habit in the control group were study for an abnormal chest or abnormal lung function by X-rays and following excluded from the control population set [7, 8]. Additionally, detailed questionnaire designed by Indian Council of Medical Research (ICMR), New Delhi which included basic information, including age, gender, disease history, history of cigarette smoking and so on was also collected from each population set (Table 1), and provided the respective detailed inform consent during this study. All participants were recruited from the hospitals nearby region of Chandigarh in north India. The present study was approved by the Human patients’ ethical committee of Government Medical College and Hospital (GMCH), Sector 32, Chandigarh, India [7, 8]. 

**Genomic** **DNA** **isolation and genotyping: **Under sterile conditions, 2 mL of peripheral blood was collected from respective population set and subjected to genomic DNA extraction from the lymphocytes by phenol/chloroform DNA extraction method at pH 7.4, as given by Sambrook *et al.* [9]. The region containing the I/D polymorphism on intron 16 of the *ACE* was amplified using the forward primers i.e. 5'-GCCCTGCAGGTGTCTGCAGCATGT-3' and reverse primer i.e. 5'-GATGGCTCTCCCCGCCTTGTCTC -3' synthesized from Sigma Aldrich, India [10]. 

**Statistical analysis**: The distribution of *ACE* I/D gene polymorphisms frequency and the relation of demographic variables among cases and controls were statistically evaluated along with by applying SPSS, version 11.5. The statistical relevance of relationship between risk factor and susceptibility of disease was defined by odds ratio (OR) with its 95% Confidence Interval (95% CI). Furthermore, Hardy-Weinberg equilibrium was also estimated with the aid of Chi-squared (χ^2^) test while significant P<0.05 was considered.

## RESULTS AND DISCUSSION

Table 1 show gender, smoking habit and age of the participants. There were significant differences between cases and controls for gender and age of the aprticiapnts. 

Particiapnts were classified into three major habit groups; (i) smokers and non-smokers, (ii) users and non-users of tobacco and (iii) users and non-users of alcohol. However, only smokers and non-smokers patients were selected in the present study. It was found that 63% and 50.5% of the patients and controls were smokers, respectively. It means that there was a significant association between smokers and risk of COPD (OR=1.67, 95% CI=1.12-2.48, *P=*0.012).

**Table 1:** Demographic characteristic of cases and controls

**Characteristics**	**Control**	**COPD Patients**	**OR (95% CI)**	*** P***
Female	86	66	1.0	-
Male	114	134	1.53(1.02-2.29)	0.039
Non-smokers	99	74	1.0	-
Smokers	101	126	1.67(1.12-2.48)	0.012
Average age (±SD)	44.2 ± 14.4	52.1 ± 15.5	-	<0.001

In case of *ACE* (intron 16, I/D) polymorphism*, *the frequency of homozygous (II) genotype was higher in case of controls (22%) than in cases (18.5%), whereas the frequency of heterozygous (ID) genotype in controls was 45% and in cases it was 22%. The frequency of homozygous (DD) genotype was more in cases (59.5%) than in controls (33%). The genotypes showed significant association with the risk of COPD. The DD genotype, significantly inceasered the risk of COPD (OR=2.14, 95% CI=1.22-3.78, P=0.006). But in case of ID genotype, no substantial association was recorded (Table 2). Among smokers, DD genotype showed statistically significant association with increased risk of COPD. Amongst non-smokers, genotypes were not associated with the risk of COPD (Table 2). 

**Table 2:** Association between the *ACE* I/D genetic polymorphism and risk of COPD

**Genotypes**	**Cases (200)**	**Controls (200)**	**OR (95% CI)**	**P**
Total				
II	37	44	1.0	-
ID	44	90	0.58 (0.32-1.07)	0.082
DD	119	66	2.14 (1.22-3.78)	0.006
				
Smokers				
II	26	30	1.0	-
ID	22	42	0.60 (0.27-1.35)	0.246
DD	78	29	3.10 (1.50-6.47)	0.001
				
Non-smokers				
II	11	14	1.0	-
ID	22	48	0.58 (0.21-1.65)	0.374
DD	41	37	1.41 (0.52-3.84)	0.606

Previoulsy the association between this polymorphism and other diseases such as end stage renal disease have been reported [11]. Present results revealed the positive association between the DD genotype and the risk of COPD. Among smokers, DD genotype showed statistically significant association with increased risk of COPD. 

In contrast to our findings, several studies did not show any positive association between the *ACE* I/D polymorphism and the risk of COPD [12]. In the same way, a meta-analyses also concluded the same result as the previous study showed significant association between *ACE* polymorphism and risk of COPD in Caucasian and Asian population [13]. 

This study has limitations such as (1) small sample size, (2) complicated etiology of COPD (3) fickle epidemiological criteria and (4) quantification the levels of ACE. 
